# Intracellular Calcium as a Regulator of Polarization and Target Reprogramming of Macrophages

**DOI:** 10.3390/ijms262411901

**Published:** 2025-12-10

**Authors:** Marina Y. Pogonyalova, Daniil Y. Popov, Andrey Y. Vinokurov

**Affiliations:** Cell Physiology and Pathology Laboratory, Orel State University, Orel 302026, Russia; mpogonalova@gmail.com (M.Y.P.); rennda@yandex.ru (D.Y.P.)

**Keywords:** macrophages, polarization, calcium homeostasis, calcium signaling, reprogramming

## Abstract

Macrophage metabolic plasticity providing their polarization towards classically (M1) or alternatively (M2) activated cells is an important element of the initiation, development, and resolving or inflammation-linked pathologies. The prevalence of M1 or M2 types of macrophages during different stages of diseases supports increased inflammation and phagocytosis or tissue repair, respectively. An imbalance leading to a shift toward an M1- or M2-dominant state is associated with a chronic pathological process. This characterizes the regulation of macrophage phenotypes as a prospective strategy in the treatment of various diseases and makes it relevant to a deep understanding of the mechanisms defining cell polarization. According to the central role of calcium signaling in cell metabolism, changes in calcium homeostasis are closely linked to the regulation of polarization. The exact balance between calcium flows across plasma and intracellular membranes provided by a number of receptors and channels, as well as the differences in the calcium-buffering capability of endoplasmic reticulum and mitochondria, are able to influence macrophage polarization towards an M1 or M2 phenotype. This review focuses on the role of the calcium homeostasis system in macrophage functionality and calcium-induced changes in macrophage metabolism that forms the basis of target disease therapy.

## 1. Introduction

Calcium intracellular signaling system is one of the key players of cell metabolism: physiological calcium concentration changes are linked with the regulation of mitochondrial bioenergetics, lipid metabolism, cytoskeletal modifications, and pathological changes leading to cell death [1,2,3]. The gradients of calcium ions (Ca^2+^) concentration relative to the plasma and intracellular membranes are the signs of healthy cells. A steady-state cytosolic calcium concentration ([Ca^2+^]i) (about 100 nM) is 10,000-fold lower compared to the extracellular space, while cell stimulation leads to a 10–100-fold [Ca^2+^]i increase [4]. The capability of recovery to initial [Ca^2+^]i, preventing cell damage, is provided by the calcium homeostasis system. It is based on the balance between Ca^2+^ flows through membranes and the Ca-buffering capability of the cytosol and main intracellular depos–endoplasmic reticulum (ER), mitochondria, and lysosomes. [Ca^2+^]i increase is provided by extracellular Ca^2+^ entry though a number of channels, as well as Ca^2+^ release, mainly from ER [5,6,7,8]. The last is closely linked to cell membrane metabotropic purine receptors P2Y (P2YRs), which are coupled with G-proteins (D_q_/G_11_, G_i_/D_0_, G_12_/G_13_ or G_S_ depending on receptor type) [9]. ER calcium release is mainly mediated by the activation of phospholipase C (PLC) by P2Y1R and P2Y6R and the production of inositol triphosphate (IP3), affecting IP3 receptors on the ER membrane [10]. [Ca^2+^]i decrease is an energy-consuming process of calcium transport into extracellular space through plasma membrane Ca^2+^-ATPase or into the ER through sarcoplasmic/endoplasmic reticulum Ca^2+^-ATPase (SERCA) [11,12].

Macrophages are innate immune cells that are present in virtually all tissues, where they perform various functions related to the homeostasis, development, and protection of the body [13,14,15,16]. Tissue-resident macrophages (TRMs) are usually defined as macrophages that colonize tissues during embryonic development and remain there for a long time, often capable of self-renewal in situ [17]. The type of TRM is dependent on the tissue location (Kuppfler cells in liver, Langerhans cells in skin, osteoclasts in bones, microglia in brain, etc.) [18]. In contrast, macrophage-derived macrophages (MDMs) arise from adult bone marrow hematopoietic stem cells via circulating monocytes that can enter tissues, especially during inflammation or the depletion of resident populations [13,19].

During inflammation, resident macrophages accumulate and, in some cases, are replaced by recruited macrophages derived from monocytes. The functional properties of macrophages, both in homeostasis and in disease, are determined not only by their ontogenesis but also by signals from the local tissue microenvironment [20,21,22]. This concept, that origin is independent of activation status, has become a fundamental principle in macrophage biology, emphasizing that two morphologically similar macrophages can have very different histories of origin and functional predispositions. In adulthood, circulating monocytes serve as a source of macrophages that can colonize tissues, especially in response to stimulation. It is important to understand that monocytes themselves are heterogeneous and consist of functionally distinct subgroups [23].

According to the differences in polarizing signals, the role in physiological processes and metabolic specificity macrophages are traditionally divided into two groups—M1 and M2. Classically activated macrophages (M1) are usually induced by microbial products such as lipopolysaccharide (LPS) and interferon-γ (IFN-γ), whereas alternatively activated macrophages (M2) arise in response to interleukin-4 (IL-4) and interleukin-13 (IL-13)—cytokines produced by anti-inflammatory cells (Th2-lymphocytes, innate lymphoid cells type 2) [24]. In addition, one more macrophage group includes the main tumor-infiltrating immune cells, named tumor-associated macrophages (TAMs) [25].

M1 macrophages are characterized by a high production of pro-inflammatory cytokines, including tumor necrosis factor-α (TNF-α), interleukin-1b (IL-1b), and interleukin-6 (IL-6), as well as chemokines such as CXCL9 and CXCL10 [26,27,28]. A key biochemical feature is the activation of induced nitric oxide synthase (iNOS), which leads to the formation of nitric oxide (NO) and reactive oxygen species (ROS), which provide antimicrobial and antitumor activity [29]. From the point of view of metabolism, M1 polarization is associated with increased glycolysis and impaired activity of the tricarboxylic acid (TCA) cycle. The accumulation of metabolites such as succinate stabilizes hypoxia-induced factor-1a, further enhancing the expression of pro-inflammatory genes [30]. At the same time, fatty acid oxidation and the oxidative phosphorylation (OXPHOS) of mitochondria are suppressed, which leads to metabolic reprogramming with functional specialization [31]. Functionally, M1 macrophages play a central role in protecting the body from bacteria, viruses, and protozoa, exerting a bactericidal effect and coordinating the involvement of other immune cells. In the context of pathology, they contribute to acute inflammation and tissue damage when their activity is prolonged [32].

Extensive studies identified variability in alternatively polarized macrophages that allow us to divide them into four main groups—M2a, M2b, M2c, and M2d [33,34,35]. While the first three groups are associated with tissue repair and healing with anti-inflammatory release, the fourth group is responsible for scar formation and fibrosis releasing as anti- and pro-inflammatory cytokines [33]. M2 macrophages are characterized by the expression of common receptors such as CD206 (C-Type Mannose Receptor 1) and CD163 (Hemoglobin–Haptoglobin Scavenger Receptor), as well as enzymatic markers such as arginase-1 [35]. In contrast to the pro-inflammatory functions of M1 cells, M2 macrophages contribute to the elimination of inflammation and the restoration of tissue homeostasis, and also play a central role in angiogenesis and wound healing [36,37].

The metabolic profile of M2 macrophages differs significantly from that of M1 cells. Instead of aerobic glycolysis, M2 polarization is associated with the increased activity of TCA and OXPHOS, and enhanced fatty acid oxidation [38]. This metabolic orientation provides the body with energy and biosynthetic precursors necessary to maintain tissue repair functions.

## 2. Macrophage Polarization in Progression and Resolution of the Pathologies

Macrophage polarization is an evolutionarily proven instrument in inflammation-linked disease progression and resolution [39,40,41]. While M1 cells are responsible for initiating and sustaining inflammation through releasing pro-inflammatory cytokines and the production of ROS, M2 cells have an anti-inflammation profile including wound healing and tissue repair [40]. So, disease resolution is a result of a well-orchestrated dynamic balance between macrophage types on different stages of the pathology, which is determined by the processes of cell polarization and reprogramming [42,43,44]. Imbalance in macrophages ratio and a persistent shift toward an M1- or M2-dominant state contributes to the pathogenesis of multiple diseases.

The prevalence of M1 macrophages is a sign of chronic inflammation revealed in several pathologies. In atherosclerosis, an increase in pro-inflammatory cells is associated with the growth and instability of plaques and foam cell necrosis with disturbances in the apoptosis-based mechanism of defective cell removal [45]. A widely used model of atherosclerosis (Apolipoprotein E double knockout mice, ApoE-KO) showed that early atherosclerotic plaques were enriched with M2-like macrophages, which promoted the proliferation of smooth muscle cells in vitro, whereas, in elderly mice, a transition from an M2 to M1 phenotype was observed, which is due to the repolarization of macrophages in the lesion. A correlation of lesion progression with the dominance of the M1 phenotype was also determined [46]. High levels of pro-inflammatory cytokine secretion by M1 is a part of the acute or chronic development of autoimmune diseases, including rheumatoid arthritis, systemic lupus erythematosus, Behcet’s disease, multiple sclerosis, lupus nephritis, and multiple sclerosis [34,47,48]. An increased M1 and decreased M2 polarization macrophage level is detected in obesity and type 2 diabetes, indicating that they are chronic inflammatory pathologies [49,50]. Interestingly, this imbalance, coupled with glycemia, leads to the severe development of some other pathologies, including infections [51]. Yao et al., using a Parkinson’s disease (PD) model, C57BL/6 mice treated with methyl-4-phenyl-1,2,3,6-tetrahydropyridine, showed that the microglial activation of the M1 phenotype increases and M2 is suppressed during aging, which correlates with more severe neurodegeneration [52].

M2 macrophage-associated pathology progression is mostly linked with the ability of this type of macrophage to increase cell proliferation, stimulating fibrosis processes and scar formation [53,54,55,56,57]. The infiltration of tumors with M2 macrophages, as well as TAMs with an M2-like phenotype, is responsible for the increased proliferation, migration ability, and epithelial–mesenchymal transition of cancer cells [53,54,55,58,59,60], making macrophages a target in complex cancer therapy. Fibrosis stimulated by M2-type cells leads to pathology progression in the narrowing of the urethral lumen [61], cardiac tissue modeling after myocardial infarction [62], pulmonary fibrosis [63,64,65], and systemic sclerosis [66].

A significant role of M1/M2 macrophage balance in health and disease makes cell polarization and reprogramming a prospective way for the therapy of different pathologies. A deep understanding of the processes regulating monocyte-to-macrophage transformation is needed for this aim. In our opinion, the homeostasis of Ca^2+^ which mediates and links many intracellular processes can be a target in macrophage phenotype regulation.

## 3. System of Calcium Homeostasis in Macrophages

**Figure 1:**
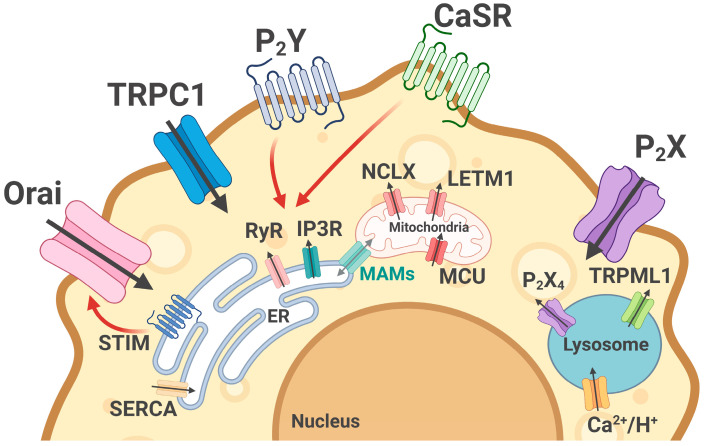
Key players of macrophage calcium homeostasis. Activation of metabotropic purinergic receptors (P2Y) and the calcium-sensing receptor (CaSR) stimulates phospholipase C–dependent inositol-1,4,5-trisphosphate (IP_3_) production, leading to Ca^2+^ release from the endoplasmic reticulum (ER) via IP_3_ receptors (IP_3_R). Ca^2+^ reuptake into the ER is maintained by SERCA pumps, ensuring restoration of basal calcium homeostasis. Store depletion activates stromal interaction molecule 1 (STIM1) and Orai channels, initiating store-operated calcium entry (SOCE). Ca^2+^ entry is also mediated by TRPC1 and ionotropic receptors purinergic (P2X). Ca^2+^ influx in mitochondrial matrix occurs mainly via the mitochondrial calcium uniporter (MCU) while efflux is linked with the function of the Na^+^/Ca^2+^ exchanger (NCLX) and (leucine zipper–EF hand-containing transmembrane protein 1) LETM1. Mitochondria-associated membranes (MAMs) facilitate ER-to-mitochondria Ca^2+^ transfer. Lysosomal channels such as P2X4 and TRPML1 further contribute to intracellular Ca^2+^ dynamics. Black arrows mean the Ca^2+^ flows, red arrows mean the influence of P2Y receptors and CaSR activation on RyR and IP3R and the effect of STIM on SOCE.

Calcium signaling plays a key role in the immune response during infections and inflammation. Calcium promotes cell survival by activating transcription factors, cell proliferation, and the development of phagolysosome fusion [6,67]. Macrophage [Ca^2+^]i regulation is provided by a well-orchestrated system of receptors and channels of plasma membrane and cell organelles (Figure 1).

Ca^2+^ influx from extracellular space is mediated by a number of channels opening to external and internal stimuli. External signal-stimulated channels are mostly presented by the ATP-dependent purinergic ionotropic receptors P2X4R and P2X7R and some members of the transient receptor potential (TRP) channel super-family (TRPC1, TRPV2, TRPM2) [68,69,70,71,72,73,74]. Despite their non-excitable character, macrophages also express some voltage-dependent channels (i.e., L-type calcium channels) [75,76].

Another way of [Ca^2+^]i increase is linked with ions released from intracellular depos, mostly from ER and lysosomes. As in other cell types, Ca^2+^ accumulation in the ER is mediated by SERCA, while ryanodine (Ry-) and inositol-3-phosphate (IP3-) receptors are responsible for Ca^2+^ release [77,78]. One of the most studied ways of ER calcium release is associated with the activation of P2YRs [68], mostly P2Y2R and P2Y6R coupled to Gq protein (although there is evidence of the presence of other types of receptors in macrophages, in particular P2Y14R) [79,80,81,82]. ER Ca^2+^ release is also linked with a lesser known inducer, i.e., bitter taste receptors (T2R), leading to a low-level calcium signals through IP3R activation [83]. ER calcium efflux explains [Ca^2+^]i’s experimentally registered oscillations, which can mostly be prevented by using IP3R or RyR antagonists or by the removal of extracellular Ca^2+^ [84,85,86,87,88,89,90].

ER Ca^2+^ reflux is closely linked with an important immune cell signaling pathway—Ca^2+^ entry through the plasma membrane via calcium release-activated channels (CRAC) [91], which is called store-operated calcium entry (SOCE) [92,93,94,95]. CRACs are composed of Orai proteins, which form the channel pore and are activated by stromal interaction molecules STIM1 and STIM2. Located in the ER membrane, STIM1 and STIM2 perform a dual function: sensing Ca^2+^ concentration in the ER and activating CRACs for ER refilling [96,97].

Despite their relatively small size, lysosomes play an important role in calcium signaling due to a wide cell distribution and, therefore, provide the possibility for local [Ca^2+^] regulation. To date, the list of channels responsible for Ca^2+^ efflux from lysosomes includes the mucolipin subfamily of transient receptor potential (in macrophages, mostly TRPML1), two-pore channels (TPC2), and ATP-gated P2X4R [98,99]. However, the mechanism of lysosome Ca^2+^ refilling is a debatable question. Due to their high acidity, Ca^2+^/H^+^ exchangers, i.e., LCI encoded by *TMEM165*, may drive pH-dependent Ca^2+^ uptake into lysosomes [98,100]. Another known way is closely linked with SOCE and ER-to-lysosomes Ca^2+^ transport [101].

While mitochondrial Ca^2+^ uptake is primarily mediated by mitochondrial calcium uniporter (MCU), which is regulated by two subunits—MICU1 and MICU2 [84]—Ca^2+^ release is linked with the Na^+^/Ca^2+^ exchanger (NCLX) and leucine zipper–EF hand-containing transmembrane protein 1 (LETM1) [102,103]. One more mechanism of mitochondrial Ca^2+^ release is linked with the short-term opening of the mitochondrial permeability transition pore (mPTP) [104,105].

No less important for mitochondrial calcium buffering is a close connection of mitochondria with ER through mitochondria-associated membranes (MAMs), forming by RyR and IP3R and mitochondrial voltage-dependent anion channels (VDAC) [106,107,108]. The close contact of ER and outer mitochondrial membrane forming MAM is provided by tethering proteins including voltage-dependent anion channels (VDAC), Mitofusin-1/2, protein tyrosine phosphatase-interacting protein-51 (PTPIP51), the Fission 1 of mitochondria and IP3R, Mitofusin-2, vesicle associated membrane protein-associated protein B (VAPB), and B cell-associated protein 31 (Bap31) of ER [109]. Among the number of functions [110,111,112], Ca^2+^ transport from the ER to the mitochondrial matrix mediated by IP3R, VDAC, and chaperone protein glucose-regulated protein 75 (GRP75) [113] play a pivotal role in mitochondria function regulation: a moderate load of mitochondrial matrix with Ca^2+^ stimulates some of the enzymes (pyruvate dehydrogenase, α-ketoglutarate dehydrogenase, isocitrate dehydrogenase), leading to metabolism enhancement, an increase in mitochondrial membrane potential, and ATP production [114]. However, extreme mitochondrial Ca^2+^ concentration increase leads to enhanced ROS production, the collapse of the electron transport chain function which is a trigger of prolonged mPTP opening and cell apoptosis [115,116].

## 4. Calcium Signaling and Macrophage Functions

The calcium homeostasis system determinates macrophage functionality. In monocytes and macrophages, P2Y2Rs play an important chemotactic role in the detection of apoptotic cells releasing ATP [117]. A study on the human monocyte cell line THP-1 showed that treatment of the cells with LPS induced rapid ATP release from the cells, which was blocked by the knockdown of SLC17A9 (vesicular nucleotide transporter, VNUT), which is responsible for ATP exocytosis [82,118]. ATP release via exocytosis also activates P2Y11R, leading to macrophage activation and cytokine release [82]. At the same time, the elevation of [Ca^2+^]i coupled with the activation of G proteins is responsible for the fusion of docked macrophage vesicles and the regulation of macrophage secretion [119]. Furthermore, a role for the G protein-coupled calcium-sensing receptor (CaSR), associated with cardiovascular diseases, has been shown [120]. Elevated extracellular calcium levels can activate CaSR, initiate the corresponding ion channels, and promote calcium entry into the cell. This, in turn, leads to ER calcium release, stimulates inflammasome assembly, activates the effector protein caspase-1, and, after maturation, the release of the proinflammatory cytokine IL-1β [121]. STIM1 has been reported to be essential for FcγR-mediated calcium entry, phagocytosis, inflammatory cytokine production, and autoimmune inflammation [122], which shows a possible role of CaSR in macrophage SOCE. Disruption in lysosomal calcium regulation was linked with IL-1β release, indicating lysosomes as a player in the priming and assembly phase of NLRP3 inflammasome development [123]. Cell injury mediated by extracellular Ca^2+^ influx was shown in silica-exposed alveolar macrophages [124]. The same toxicity model with [Ca^2+^]i increase led to irreversible injury of mouse macrophage-like cell line P388D_1_, regardless of mitochondrial uncoupling or ATP content decrease [125].

Although calcium-dependence of different membrane fusion processes (i.e., release of neurotransmitters in brain cells or insulin in pancreatic beta cells) [126,127] is well-known, the mechanism of the link between Ca-signals and phagocytosis in macrophages has been discussed for a long time [128]. It was shown that, unlike neutrophils, phagosome–lysosome fusion in macrophages is Ca-independent and develops even in very low calcium concentrations [129]. The same results were observed in a model of IgG-coated erythrocytes [130], even in the case of M1-polarized macrophages [131]. Also, no change in the internalization of non-functionalized polystyrene beads in different extracellular calcium concentrations, as well after SERCA inhibitor thapsigargin action, was detected by E. Diler et al. [132]. Nevertheless, the activation and development of phagocytosis is accompanied by calcium signalization, showing its regulation role in the processes after phagosome formation. [Ca^2+^]i increase was detected as a result of FcγR receptor activation and linked with ions released from the ER [130,131,133]. In addition, spontaneous and transient changes in calcium activity in Langerhans cells is an important mechanism of skin renewal through the engulfment and phagocytosis of damaged cells, like keratinocytes [134].

Phagosomal calcium flux associated with macrophage inositol-requiring enzyme 1α activation enhances fungicidal activity by promoting phagosome maturation [135]. The efficiency of phagocytosis was also linked with ATP-based calcium signaling mediated by P2X4R and P2X7R [136]. It is noteworthy that the inhibition of calcium signaling leading to the alteration of phagosome–lysosome fusion promoted intracellular mycobacterial tuberculosis survival that was inversed by ionomycin application [137]. Calcium signals, both from extra- and intracellular sources, was required for the recruitment of Golgi-derived vesicles to the sites of different cargo uptakes and effective phagocytosis [138]. Ca-release during the fusion of lysosomes and plasma membrane is a part of the mechanism of large extracellular particle phagocytosis mediated by the activation of the big conductance Ca^2+^-activated potassium channel [139]. The importance of Ca^2+^ for NLRP3 inflammasome activation also defines the great role of intracellular calcium depos in phagocytosis. For example, MCU-mediated Ca^2+^ uptake attenuates phagosomal membrane repair and promotes NLRP3 inflammatory response [140]. As was shown by S. Tedesco et al., MCU knockdown led to phagocytosis inhibition associated with the reduced M2-polarizaton [141]. The close relationship between NLRP3 inflammasome activation and ER calcium release associated with ATP stimulation and mediated by C/EPB homologous protein was shown by T. Murakami et al. [142]. Ca^2+^ release from the intracellular store linked with T2R activation led to NO production and enhanced phagocytosis through cGMP and cAMP forming [83].

## 5. Calcium Homeostasis: M1/M2 Macrophage Specificity

**Figure 2:**
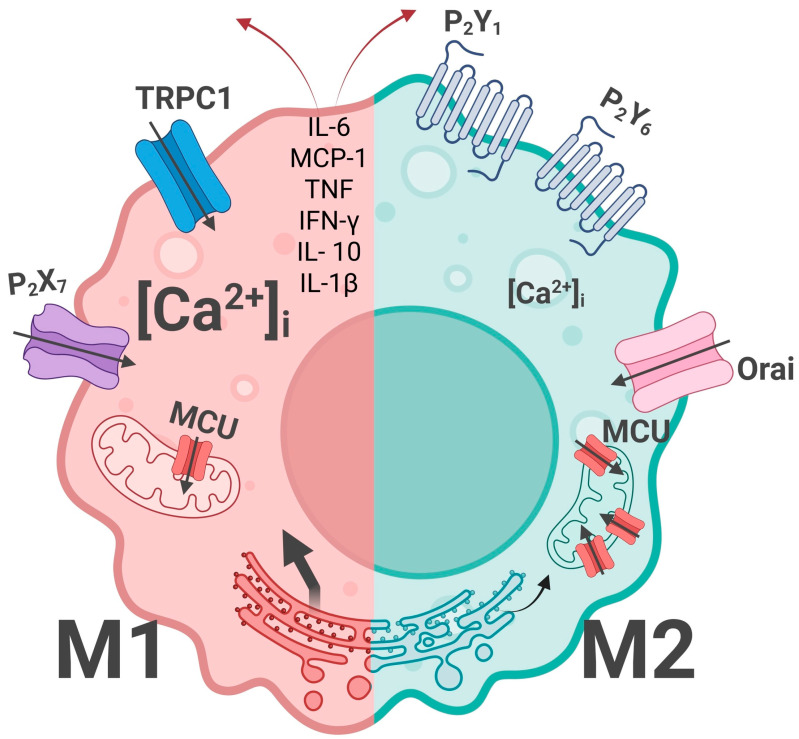
Differences in maintaining calcium homeostasis in M1 and M2 macrophages. Macrophages with pro-inflammatory effects are characterized by higher basal calcium levels compared to M2-type cells, which is associated with a decrease in mitochondrial Ca^2+^ buffering properties, including decreased mitochondrial calcium uniporter (MCU) expression. High level of calcium in M1 macrophages leads to the release of various inflammatory cytokines. Transient receptor potential canonical 1 (TRPC1) is also necessary for the polarization of macrophages towards the M1 inflammatory phenotype, while Orai channels, as well as purinergic receptors (P2Y1R and P2Y6R) are more strongly expressed in M2 macrophages. Ionotropic receptors (P2X7) on the other hand, reduce the polarization of macrophages towards M2. Black arrows mean the Ca^2+^ flows, red arrows mean cytokines release.

Considering the role of Ca^2+^ in macrophage polarization, it is important to distinguish the difference in basal (steady-state) concentration and stimulation-based change which determine M1 or M2 macrophage calcium specificity (Figure 2).

LPS and IFN-γ leading to M1-like macrophage polarization were responsible for chronic basal [Ca^2+^]i increase, leading to a high production of IL-6, MCP-1, TNF, IFN-γ, IL-10, and IL-1β [95]. The same results were obtained for TRMs (microglia), where chronic basal [Ca^2+^]i elevation is a factor of LPS-stimulated NO production and the release of certain chemokines and cytokines indicates pro-inflammatory conditions [143]. Increased inflammation and decreased bacterial colonization during Helicobacter pylori was also revealed in a *Trpm2*^−/−^ mice model exhibiting macrophage calcium overload and enhanced NADPH oxidase activity [70]. One of the reasons for the increased [Ca^2+^]i influence on macrophage polarization is linked with subsequent mitochondrial disfunction linked with calcium overload and activating the pro-inflammatory phenotype with enhanced macrophage glycolysis [144], elevated ROS production, and impairment of the ability of mitochondria to buffer intracellular calcium oscillations [145].

According to experimental data, an increase in basal [Ca^2+^]i leading to preferential M1 polarization may be a sequence of different processes. One of them is SOCE, whose inhibition significantly decreases the release of pro-inflammatory cytokines [95]. Another source of elevated [Ca^2+^]i is ER calcium release. Its inhibiting also caused an impairment in macrophage recruitment and pro-inflammatory phenotype in a model of Zebrafish wound healing [146]. However, it is important to note that ER stress associated with decreased Ca^2+^ influx through the Orai-TRPC1-STIM1 complex was linked with a promoted M1 phenotype and increased phagocytosis [147]. One more cause of elevated cytosolic [Ca^2+^] leading to inflammaging and linked with age is a time-dependent decrease in MCU and MICU1 expression decreasing mitochondrial calcium uptake [84]. TRPC1 deficiency prevents macrophages from producing IFN-γ- or Klebsiella pneumonia infection-induced M1 inflammatory mediators [148]. This means that TRPC1 expression influences macrophage plasticity, and TRPC1-mediated calcium influx is necessary for macrophage polarization toward the M1 inflammatory phenotype [148]. This conclusion is confirmed by other authors [149], who have found that, while cells with an M1 phenotype are mostly dependent on TRPC1 Ca-entry in naïve or M2 macrophages, this process is preferentially associated with Orai channels.

The level of stimulated calcium signal is no less important for macrophage polarization. Purinergic receptors are of interest in this aspect. Comparing P2R profiles, J. Merz et al. have concluded that the principle difference between P2-based calcium signaling in M1 and M2 macrophages is the increased [Ca^2+^]i change in anti-inflammatory cells after ADP application, which is mostly linked with P2Y1R and P2Y6R receptors [81]. However, the polarizing role of P2XRs is also known. While P2X4Ractivation led to the transition from acute to persistent inflammation through the phosphorylation of p38MAPK, physical exercise-based PPARγ increased activity was associated with the reverse effect and inflammation resolve [150]. P2X7R, which is highly expressed in immune cells, plays a significant but contradictory role in macrophage plasticity. On the one hand, a deficiency in P2X7R, which is highly expressed in TAMs, decreases M2-like macrophage polarization, showing the way for lung cancer therapy [71]. In addition, the increased expression of this receptor was linked with prevalent M2 macrophage polarization in a study of macrophages’ role in chronic heart allograph rejection [151]. On the other hand, during ATP-influenced spontaneous M2 polarization, P2X7Rlevel downregulated [152]. Although the activation of P2X7R induces the assembly of NLRP3 inflammasome and the release of pro-inflammatory cytokines, it is also linked with inflammation resolution through the release of anti-inflammatory proteins [153]. It is interesting that P2X7R plays an important role in macrophage M1-to-M2 reprogramming [73]. While in M1 cells the action of ATP on the receptor leads to the release of pro-inflammatory IL-1β and NLRP3 inflammasome activation, in intermediate states (during M1-to-M2 transition) the same ATP influence uncouples NLRP3 inflammasome activation and inhibits IL-1β release. It should be noted that the last is not inherent to M2 macrophages’ state. The dual role of P2X7Rin inflammation was also shown by E. Townsend et al. in a study of alveolar macrophages obtained from patients with asthma. It was found that P2X7R signaling is linked with the release of pro-inflammatory or pro-resolving lipid mediators [154].

The M1 macrophage polarization route is closely linked to intracellular [Ca^2+^]i oscillations. The LPS stimulation effect was significantly decreased after the inhibition of FcRγ induced calcium oscillations in bone marrow macrophages [85]. P. Hanley et al. have shown that, stimulated by moderate ATP concentration (10 μM), calcium oscillations mediated by P2Y2R and PLC activation lead to an IL-6 transcription increase [86]. In a study [78], the knockout of TRIM family protein MG53 was linked with RyR-linked Ca^2+^ oscillations and subsequent interferon-β release. The effect of bioceramics for treating bones’ effect on the subsequent rise and decline in [Ca^2+^]i and calcium oscillations was shown by J. Zhao [87]. The first stage of bioceramics degradation led to an increase in [Ca^2+^]i and the stimulation of M1 macrophage polarization, but a decrease in extracellular calcium promotes M1-to-M2 transition.

The decreased Ca^2+^ influx caused by the blockade of the K+ Kir2.1 channel or membrane depolarization was responsible for the suppression of M1-like but increased M2-like macrophage polarization through the CaMK II/ERK/NF-κB pathway, indicating Kir2.1 as a potential target in modulating inflammatory processes [155].

The dependence of macrophage polarization on extracellular Ca-signaling can be illustrated by the study of the cells of patients with rheumatoid arthritis [156], where calcium-macrophages with excessive, constitutive SPP1/osteopontin production and a strong pro-inflammatory cytokine response due to CaSR activation were identified.

The role of mPTP, which is calcium-dependent in macrophage phenotype regulation, is of interest. The inhibition of mPTP formation by cyclosporine A significantly decreases cytoplasmic mitochondrial DNA (mtDNA) release and the inflammatory response in macrophages deficient in mitochondrial membrane protein prohibitin 1 [157]. The inhibition of cyclophilin D, one of the mPTP components, also alleviated links with thyroid-stimulating hormone receptor pro-inflammatory polarization and oxidative stress in primary peritoneal macrophages and RAW264.7 cells [158]. The positive correlation of the LPS-based macrophage M1 polarization route with mPTP increase was shown in vitro in a culture of alveolar macrophages [159].

The data of the macrophage type specificity of MAMs are rather contradictory. On the one hand, it is shown that MAMs play an important role in inflammaging: while inactivated NRLP3-inflammasome is linked with the ER but after activation it migrates to the contact site of the ER and mitochondria [108,160]. In addition, a reduced MAMs formation is linked with improved OXPHOS and the normalization of mitochondrial Ca^2+^ homeostasis was shown in the model of the deficiency of connexin 43, which promotes M1 macrophage polarization [161]. On the other hand, according to the results of IP3R–VDAC interaction measurements, LPS+IFN-γ stimulated macrophages have a significantly reduced level of MAMs [162]. Due to the responsibility of IP3R–VDAC interaction for ER-to-mitochondria Ca^2+^ transport, there is a decrease in the above-mentioned stimulation of mitochondrial bioenergetics that is a feature of pro-inflammatory macrophages.

## 6. Calcium Homeostasis Regulation in Macrophage Reprogramming: Perspectives for Therapy

Macrophage metabolic plasticity makes it possible to use different approaches for cell polarization regulation, which seems to be a prospective way for the resolving of macrophage-linked pathologies of different origins. For example, Guiducci et al. reported that the cell culture of MCA38 (H-2b), colon carcinoma obtained from primary cultures, switches form of infiltrating macrophages from M2- to M1-like (high levels of IL-12/iNOS), causing hemorrhagic necrosis and tumor destruction [163]. Hagemann et al. report that NF-κB (IKKβ) inhibition, specifically in TAMs, reprograms them to an M1 cytotoxic profile (IL-12^high^/Arg1^low^/NOS2^high^), restores antitumor activity, and regresses advanced ovarian tumors in vivo [164]. Sanson et al. showed the possibility of a reverse transition from M1 to M2 in mouse atherosclerotic plaque when exposed to HDL through STAT6 phosphorylation, thereby causing plaque regression [165]. In a PD model, rats with 6-OHDA damage treated with glucocorticoids targeting CD163 (a marker of M2 macrophages) shifted brain myeloid cells towards anti-inflammatory (M2-like) phenotypes and protected dopaminergic neurons of the substantia nigra [166].

According to the above analysis of the literature, calcium homeostasis regulation is one of the instruments for the polarization route regulation or remodeling of cell phenotypes as a strategy for the release of pathologies linked with macrophage type imbalance.

Some of the known studies are based on the regulation of extracellular Ca^2+^ concentration, possibly influencing CaSR and CaSR-linked intracellular calcium signalization. An alginate-based calcium release system reinforcing the M1-macrophage phenotype and increasing pro-inflammatory cytokines was studied by Y. Abib et al. [167]. It is interesting that alginate-only was responsible for the formation of pro-repair ability and the increase in the phagocytic activity of cells. The positive correlation of cell microenvironment ions (including Ca^2+^) concentration with a shift toward M1-like macrophage phenotypes was shown in a study of biocompatible materials [168,169]. The sequential regulation of macrophage phenotypes by the release of Ca^2+^ and Zn^2+^ from the hydrogel of acrylate-modified engineered protein with oxidized sodium alginate was used for the promotion of M1 polarization for infection inhibition, and then M2 polarization for the promotion of osteogenic differentiation during the restoration of infected bone defects [170].

The other findings are directly linked with intracellular [Ca^2+^]i regulation. Q. Zhiao et al. suggested lysosomal pH-sensitive ZIF-8 nanoparticles releasing ions (Ca^2+^ and Zn^2+^) from a metal–organic matrix into lysosomes, leading to the restoration of impaired phagocytosis [171]. The usage of calcium-chelating mesoporous silica nanoparticles demonstrated the reverse of the pro-inflammatory phenotype of mouse bone marrow-derived macrophages due to [Ca^2+^]i regulation and the prevention of mitochondrial Ca^2+^ overload that can be used for the osteoarthritis therapy [145]. Near-infrared-regulating nanoparticles with the ability to increase or decrease [Ca^2+^]i were shown for predominant macrophage polarization into M1- or M2-type, respectively [172]. The possibility of macrophage function improvement in chronic obstructive pulmonary disease by a change in calcium homeostasis was shown in the model of MDMs incubated with *Haemophilus influenzae* [173]. An increase in external Ca^2+^ concentration improved the phagocytosis, cytokine secretion, and cell surface expression of bacterial recognition receptors. The activation of transient receptor potential vanilloid type 4 (TRPV4), leading to a [Ca^2+^]i increase, triggered the activation of the transcription factor CREB and an increased production of IL-10, associated with the anti-inflammatory macrophages phenotype [174]. The inhibition of osteoclastogenesis in rheumatoid arthritis therapy by cytotoxic T-lymphocyte antigen 4-immunoglobulin was linked with decreased [Ca^2+^]i oscillations and LPS-induced bone resorption [85].

Since mitochondria are one of the key regulators of cell metabolism and, therefore, macrophage plasticity [145,175,176], and also play an important role in calcium homeostasis regulation, these organelles are the focus of macrophage-regulation studies. While some of the studies are directed towards complex macrophage metabolism change, the others are linked with mitochondrial calcium buffering properties. The inhibition or knockdown of MCU preventing mitochondria calcium uptake may be used for significantly reducing M2-like polarization without changing the level of M1-macrophages that can be used in the therapy of pathologies with stimulated cell proliferation or fibrosis [116]. It was demonstrated that inhibiting ER–mitochondria coupling with reducing mitochondrial calcium overload and shifting macrophage metabolism from glycolysis to OXPHOS can be achieved by amorphous calcium zinc phosphate nanoparticles [177]. The dominant-negative mitochondrial calcium uniporter subunit regulation is capable of M1-to-M2 reprogramming in macrophages, infiltrating damaged skeletal muscle, which can be useful in muscle regeneration therapy [178]. According to the significant role of mPTP in mitochondrial metabolism and the release of pro-inflammatory and pro-apoptotic factors in cytosol, its regulation is an expected target in macrophage phenotype regulation for therapy [157,179,180]. The anti-inflammatory effect linked with mPTP inhibition was shown in a study of tanshinone IIA, an active ingredient of *Salvia miltiorrhiza* [159].

## 7. Conclusions

The system of intracellular calcium homeostasis plays an important role in determining macrophage phenotype and the mechanisms of cell reprogramming. The close relationship of extracellular Ca^2+^ with intracellular calcium depos mediated by numerous receptors and transport systems provides macrophage functionality and the role of switching between M1- and M2-states in the development and resolving of pathologies. Disturbance in this well-balanced system leads to a pathological prevalence of a definite cell type with the development of a chronic inflammation process or undesired cell proliferation and fibrosis. Different macrophage types are characterized by specific calcium-linked processes and regulators that make it possible to shift cell type in a desired direction by the regulation of steady-state calcium concentration or the character of its stimulated change.

## Data Availability

No new data were created or analyzed in this study. Data sharing is not applicable to this article.

## Abbreviations

The following abbreviations are used in this manuscript:

CaSR	Calcium-sensing receptor
CRAC	Calcium release-activated channel
ER	Endoplasmic reticulum
GPCR	G protein-coupled receptor
IP3R	Inositol-3-phosphate receptor
LPS	Lipopolysaccharide
MAMs	Mitochondria-associated membranes
MCU	Mitochondrial calcium uniporter
MDMs	Macrophage-derived macrophages
NCLX	Na^+^/Ca^2+^ exchanger
OXPHOS	Oxidative phosphorylation
PD	Parkinson’s disease
RyR	Ryanodine receptor
SOCE	Store-operated calcium entry
TAMs	Tumor-associated macrophages
TCA	Tricarboxylic acid cycle
TRMs	Tissue-resident macrophages
VDAC	voltage-dependent anion channel
VNUT	vesicular nucleotide transporter
